# Methylprednisolone Plus Low-Dose Methotrexate for Bullous Pemphigoid—A Single Center Retrospective Analysis

**DOI:** 10.3390/jcm11113193

**Published:** 2022-06-02

**Authors:** Agoritsa Gravani, Georgios Gaitanis, Panagiota Spyridonos, Ioannis Alexis, Stelios Tigas, Ioannis D. Bassukas

**Affiliations:** 1 Department of Dermatology, University General Hospital of Ioannina, 45500 Ioannina, Greece; ritsagravani@gmail.com (A.G.); ggaitan@uoi.gr (G.G.); ioannisalexis@outlook.com (I.A.); 2 Department of Skin and Venereal Diseases, Faculty of Medicine, School of Health Sciences, University of Ioannina, 45110 Ioannina, Greece; 3 Department of Medical Physics, Faculty of Medicine, School of Health Sciences, University of Ioannina, 45110 Ioannina, Greece; pspyrid@uoi.gr; 4 Department of Endocrinology, Faculty of Medicine, School of Health Sciences, University of Ioannina, 45110 Ioannina, Greece; stigas@uoi.gr

**Keywords:** bullous pemphigoid, methotrexate, glucocorticoid sparing, methylprednisolone, combination treatment, dipeptidyl peptidase-4 inhibitors

## Abstract

Monomodal systemic glucocorticoids remain the mainstay of treatment for bullous pemphigoid (BP). In this retrospective, single-arm study, we evaluated the feasibility (efficacy and tolerability) of the combination of methylprednisolone and low-dose (up to 12.5 mg/week) methotrexate (MP + MTX) for BP. At week 12, 53/55 (96.4%) patients initiated on MP + MTX during a five-year period (potential follow up time: ≥4 years) remained on treatment. At this time-point, BP remission was achieved in all compliant patients (including *n* = 24 cases of dipeptidyl peptidase-4 inhibitors-associated BP; 12-week remission rate: 100% [95% CI: 91.9–100.0%]; mean time to remission: 29.5 days, SEM: 2.3 days) at a mean cumulative MP dose to disease control of 678.4 mg (SEM = 49.4 mg). Eight patients relapsed during follow up (10.81 [95% CI: 5.16–21.72] relapses/100 person years, py), and seven manifested a severe adverse event (6.80 [95% CI: 3.00–14.28] severe adverse events/100 py); however, 73.4% (±7.9%) had suffered neither a relapse nor a SAE at the three-years follow up. Continuing low dose MP intake (≤8 mg/day) beyond week 12 in combination with MTX minimized the risk of a feasibility limiting event (*p* = 0.013). Conclusively, the combination of methylprednisolone with methotrexate is a promising, safe, and efficient modality for BP patients, which enables rapid glucocorticoid tapering.

## 1. Introduction

Bullous pemphigoid (BP) is an acquired autoimmune blistering skin disease that preferentially affects older individuals [1]. It appears as intensely pruritic blisters, which can break and leave large, denuded areas of skin, with a resulting risk for superinfection. Monomodal systemic glucocorticoids remain the cornerstone of initial BP treatment. Thus, the administration of a daily prednisone equivalent dose of 0.50–0.75 mg/kg of one’s body weight is widely recommended as the first line treatment until stable disease remission is achieved. The suggested tapering is at fortnightly reduction steps of 1/4 to 1/3 of the last daily dose [2,3,4,5]. However, the prolonged use of systemic glucocorticoids can lead to serious, potentially fatal, adverse events, particularly in the typically older patients with BP, a population of generally frail patients at a higher risk for multimorbidity and consequent polypharmacy [6,7,8,9]. Therefore, therapeutic strategies that limit the use of glucocorticoids are required to optimize the management of BP patients [2]. Methotrexate (MTX) is a widely used immunomodulatory agent with antiproliferative and anti-inflammatory properties [10]. It has been used in dermatology since 1955, initially as a treatment for psoriasis, and since then, for various other chronic inflammatory diseases with autoimmune characteristics. [11] Treatment of BP with MTX was initially described in 1969 [12], and subsequently, a retrospective analysis of 34 patients (14 comorbid for diabetes mellitus type 2, DMT2) showed that the addition of MTX effectively contributed to disease control in cases of treatment failure with ‘moderately’ high prednisone doses [13]. Since then, a few smaller studies (including an open, multicenter retrospective study) have reported satisfactory treatment effectiveness and safety data on MTX monotherapy or on MTX as an adjuvant to various combination regimens with glucocorticoids for BP [8,14,15,16,17,18,19,20,21]. However, in most of these studies, the use of MTX was restricted to patients with glucocorticoid contraindications, elderly patients with many co-morbidities, and in mild or limited BP cases [15,22]. Finally, the MTX was also employed as a modality to stabilize disease remissions achieved with topical glucocorticoids [17,21].

Based on the above evidence, MTX is currently recommended as a second-line complementary modality for BP treatment, either to enable faster glucocorticoid tapering or as an adjuvant in glucocorticoid-refractory cases [2]. In addition, MTX monotherapy is suggested for BP patients when the use of systemic glucocorticoids is limited by certain comorbidities, such as poorly controlled diabetes mellitus, severe heart failure, severe osteoporosis, or glaucoma [2,5,13,14,16]. However, to date, the rationale for the integration of MTX into the therapeutic strategy for BP has not been supported by adequate evidence-based data.

Moreover, a substantial cohort of BP patients are DMT2 patients, in which the BP is associated with the intake of the dipeptidyl peptidase-4 inhibitors (DPP-4i, gliptins) antidiabetic agents [23,24,25]. Considering the limitations of prescribing systemic glucocorticoids for these latter patients, there is also an open question of if this subgroup of BP patients responds differently to the available treatments.

The primary objective of this monocentric, retrospective, single-arm study is to report on the feasibility, i.e., the efficacy and tolerability, of the combination of fast methylprednisolone tapering with low-dose MTX as the initial therapeutic strategy for newly diagnosed BP.

## 2. Materials and Methods

***Cohort***. Institutional Review Board permission was granted (Nr.: 23/05-11-2020 (θ. 5)), and the files of patients presenting with a new diagnosis of BP in the Dermatology Department of this Hospital between 1 January 2012 and 31 December 2016 were reviewed for the identification of BP cases initiated on methylprednisolone (MP) plus methotrexate (MTX) combination treatment. The follow-up cut off was 31 December 2020, enabling a potential follow-up period of at least 4 years. As previously described [23,24], BP diagnosis was defined according to established clinical and laboratory criteria. [2] In short, BP diagnosis was based on following deductive algorithm: Given compatible clinical features, a punch biopsy for histology and direct immunofluorescence (DIF) test were conducted. BP diagnosis was confirmed if both the above laboratory tests returned results consistent with BP diagnosis. In 4 cases with a negative DIF outcome, the BP diagnosis was confirmed with an additional indirect immunofluorescence (IIF) and anti-BP180 and anti-BP230 serum titer measurement with ELISA.

***Treatment***. MP treatment was empirically initiated at a daily dose between 16 and 64 mg p.o. (in 16 mg steps, i.e., 16, 32, 48 or 64 mg), depending on the severity of the disease and the anthropometric parameters of the patient. When disease remission was achieved (for the definition of remission, see below, next paragraph), the MP dose was tapered in weekly steps of 16 mg until a daily dose of 32 mg, which was subsequently tapered in 8 mg steps down to 16 mg and in 4 mg steps thereafter down to the minimum daily dose of 4 mg. Thus, a typical tapering scheme was *64*–*48*–*32*–24–*16*–8–4 mg (potential initial MP doses in italics). MP discontinuation was considered for patients in a sustained remission status for at least 8 consecutive weeks with a daily MP dose of 4 mg. In addition, topical glucocorticoids in the form of a commercially available 0.1% mometasone furoate cream were allowed up to a quantity of 20 gr/day.

MTX was initiated within the first week of BP diagnosis in parallel with the MP intake and at a weekly dose of 5.0, 7.5 or 10.0 mg p.o., followed by 10 mg folic acid supplementation p.o. the day after. The initial MTX dose was empirically decided, based on the clinical severity of the disease. On the scheduled re-evaluation at week 4 of treatment (about one week after the administration of the third MTX dose), an increase of the initial MTX dose by 2.5 mg/week was considered for patients with persisting disease activity. MTX intake was continued thereafter at the last effective dose and, in the absence of contraindications or serious adverse events, for at least 12 months. In case of disease reactivation during the glucocorticoid dose-tapering period, the MP dose was increased to the immediately previous level [2].

Moreover, for patients with DPP4i-associated BP cases, the revision of the antiglycemic therapy was necessary considering the anticipated deregulation of glucose control under the instituted methylprednisolone treatment for BP. Therefore, DPP4i was discontinued, and the patients were initiated, if needed, with an insulin-based antidiabetic regimen under endocrinological supervision.

***Study evaluation***. The following endpoints, without weighting, were applied for the short-term evaluation of this single-arm, retrospective, feasibility study: (a) the rate of fully treatment-compliant patients to week 12 of treatment; (b) the ‘percentage of patients in remission at week 12 after treatment initiation’ (‘12-week remission rate’; see definition below); (c) the ‘time to disease control’, i.e., time from treatment onset to disease remission; and (d) the ‘cumulative MP dose during the first 12 weeks of treatment’.

The long-term feasibility of the treatment was assessed through the evaluation of the disease relapses (see definitions in next paragraph) and the severe adverse events under the scheduled treatment. The following parameters were applied: (a) The rate of feasibility restricting events as a function of follow up time and (b) the times from treatment initiation to the respective events.

‘Disease remission’ was defined as “the clinical state of ceased disease activity without new BP lesions for at least 3 consecutive days while the patient was on a daily MP dose of 8 mg”. ‘Relapse’ was defined as “disease reactivation 6 months or longer after treatment initiation in a patient who had previously achieved stable disease control for at least four weeks”. A ‘disease reactivation’ was defined as “the development of at least 3 new blisters or one large bullous lesion (≥10 cm diameter) without a tendency to heal within a week or as the exacerbation of generalized itch in a patient already in remission”. [2]

***Statistical inference***. Data were analyzed using the SPSS platform. Analysis of variance (ANOVA) or Wilcoxon Signed Ranks tests were applied to analyze continuous variables. The 95% confidence intervals of fractions were calculated using the Wald method. The Kaplan–Meier calculator with the Log Rank (Mantel–Cox) test was applied for the analysis of the ‘time to event’ data. The association between time to event and potential predictor factors was inferred with the Cox proportional hazards model. A probability level of *p* < 0.05 was accepted to indicate statistical significance.

## 3. Results

**Figure 1:**
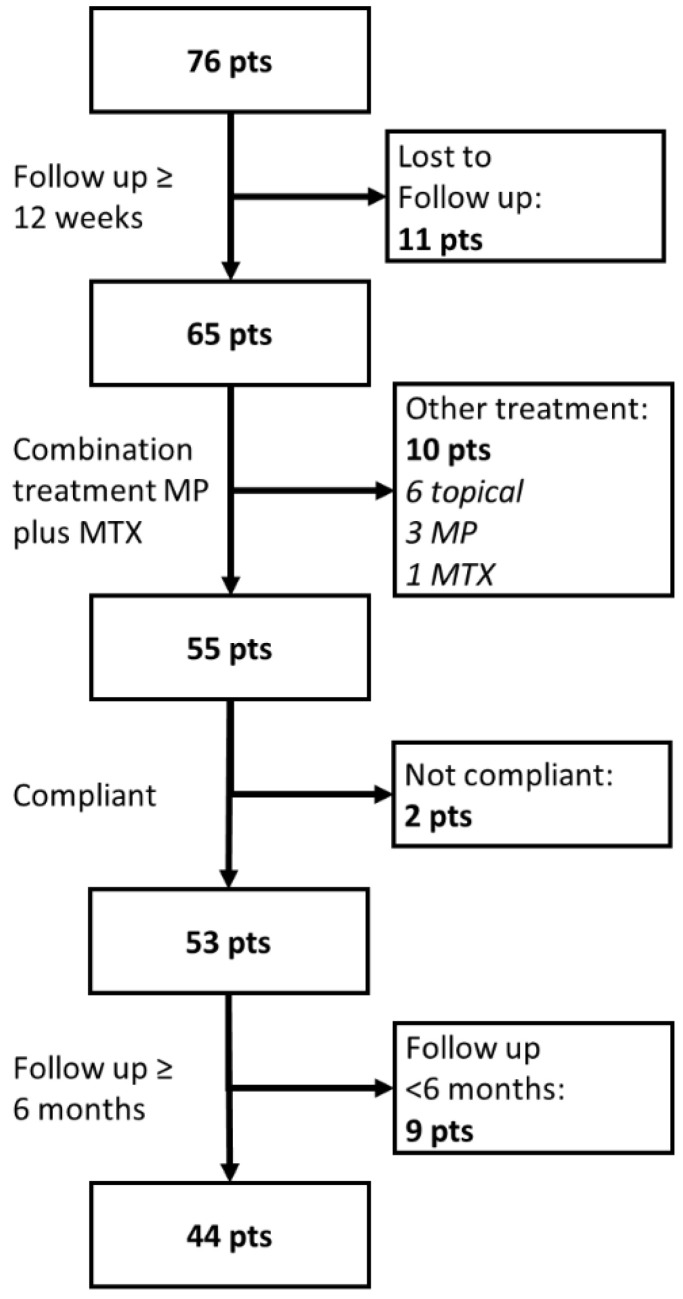
Flow chart of the inclusion strategy of the bullous pemphigoid patients. MP: methylprednisolone; MTX: methotrexate; topical: topical glucocorticoids.

***Cohort.*** A flow-chart of patient selection is displayed in Figure 1. Of the *n* = 76 patients with newly diagnosed BP, *n* = 55 patients that were initiated with the combination of systemic MP plus MTX and had a follow-up time ≥ 12 weeks were included. Twelve weeks after initiating treatment with the MP plus MTX combination scheme, 53/55 of these patients (96.4% [95% CI: 87.0–99.7%]) were fully compliant. Table 1 depicts their core demographic and disease characteristics. Twenty-one of the latter patients were male (39.6%) and 32 female (60.4%). The mean ± SEM (standard error of the mean) age of the patients was 79.0 ± 1.2 years, with 73.6% (*n* = 39) being ≥75 years old at the time of BP diagnosis. Half of the patients (*n* = 26; 49.1%) were co-morbid for DMT2, with most of them (*n* = 24 or 92.3% of DMT2 patients) being on DPP4i treatment at the time of BP diagnosis. The core demographic, treatment and disease outcome parameters of the BP patients did not differ significantly between those with and without a history of DPP4i treatment (Table 1).

***Short-term efficacy of the treatment***. For most of the patients (*n* = 38), MP treatment was initiated at 32 mg/day; the remaining patients received 16 mg (*n* = 4), 48 mg (*n* = 7), or 64 mg MP/day (*n* = 4) as an initial MP dose. The most frequently used MTX starting dose was 7.5 mg/week (*n* = 32 patients), followed by 5.0 mg (*n* = 17) and 10.0 mg (*n* = 4). Overall, about half of the patients (*n* = 25 or 47.2%) were started on the combination 32 mg MP/day plus 7.5 mg MTX/week.

**Figure 2:**
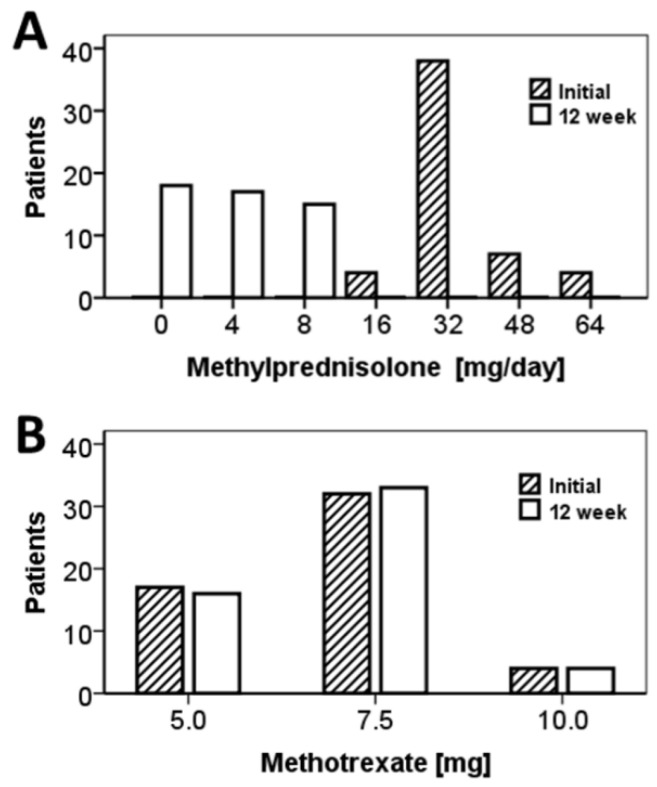
Distribution of daily MP doses (**A**) and weekly MTX doses (**B**) at treatment start and at the 12-week follow up.

BP remission was induced in all 53/55 fully compliant patients within the first 12 weeks after treatment initiation: The *per intention to treat* ‘12-week remission rate’ was 96.4% [95% CI: 87.0–99.7%] or 100% [95% CI: 91.9–100.0%] *per protocol*. The mean time and mean cumulative MP dose to disease control were 29.5 ± 2.3 days and 678.4 ± 49.4 mg, respectively. The mean cumulative MP dose of the first 12 weeks of treatment was 1009 ± 53 mg (Table 1). The mean daily MP dose at the 12-week follow up (3.96 ± 0.50 mg) was significantly lower compared to the corresponding initial dose (35.32 ± 1.52 mg; *p* < 0.001, Wilcoxon signed ranks test; Figure 2A). On the contrary, the weekly MTX dose remained quite stable during the first 12 weeks of treatment (6.84 ± 0.19 mg initially vs. 6.89 ± 0.20 mg at the 12-week follow up; *p* = 0.739, Wilcoxon signed ranks test; Figure 2B). Markedly, most patients (47/53 or about 90%) received the initial methotrexate dose throughout week 12; yet, about one-third of them (19/53, 35.8%) had already discontinued MP at that time. It is worth noting that the disease seems to be controlled more efficiently in the group of older patients with the present combination modality: Particularly, with 26.32 ± 2.07 days, the average time to disease control was significantly shorter in the age group of the older patients (≥75 years) compared to the 37.93 ± 5.87 days for patients aged < 75 (*p* = 0.022, ANOVA, Table 2).

**Figure 3:**
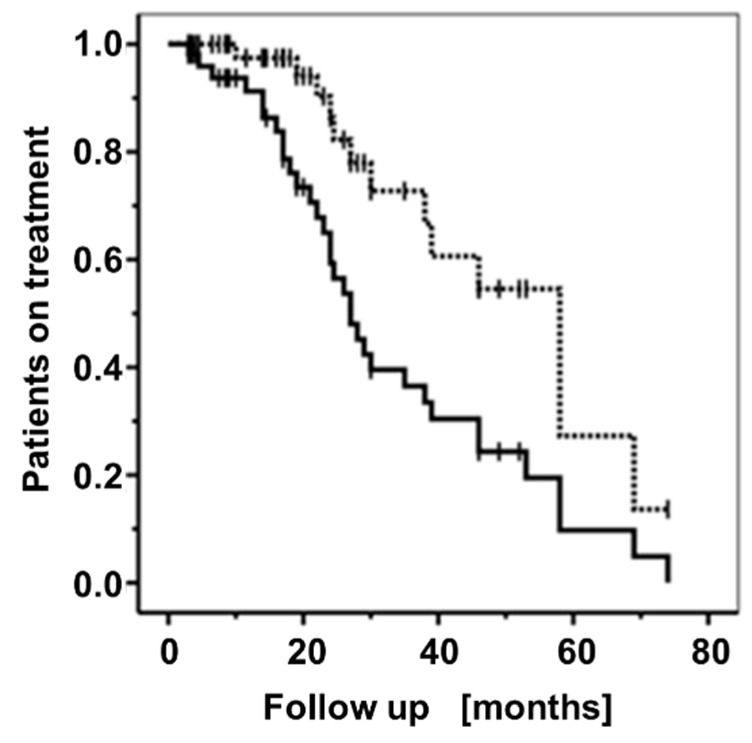
Retention of the treatment. Comparison of the rates of BP patients still receiving MP (solid line) or MTX (dotted line) as a function of time after the onset of the MP plus MTX combination therapy. Short perpendicular bars: censoring events.

***Long-term efficacy of the treatment***. The follow-up time ranged between 3 and 74 months (mean: 23.64, median: 19 months). Particularly, follow=up data for periods ≥12 months were available for 34 patients (64.5%). Patients remained on MTX treatment (median time to stop MTX: 58.0 months [95% CI: 41.5–74.5]) significantly longer than on MP (median: 27.0 months [95% CI: 21.9–32.2]; *p* = 0.02, Mantel–Cox log rank test; Figure 3).

**Figure 4:**
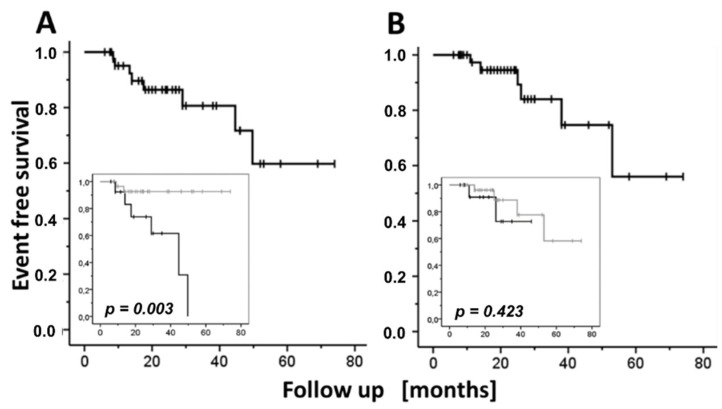
“Feasibility-limiting-event”-free survival as a function of the follow up period in months (Kaplan–Meier method). The short perpendicular bars represent censoring events. (**A**) Relapses, i.e., disease reactivations six months or longer after treatment, started in a patient who had previously achieved disease remission for at least four weeks. (**B**) Severe adverse events. Inserts: Corresponding comparisons of patients still on low-dose methylprednisolone (4 or 8 mg/day) at week 12 after the start of treatment (grey) vs. already off MP at that time (black). *p*-values: significance levels of corresponding Log-Rank tests.

Of the *n* = 44 patients with follow up periods > 6 months, eight patients relapsed between 275 and 1502 days after starting therapy (717 ± 171 days, Figure 4A). However, more than three-fourths of these patients (80.7 ± 7.7%) remained relapse-free three years after diagnosis (Table 3). The relapse rate was 10.81 per 100 person-years [95% CI: 5.16–21.72]. Except for one patient with a concurrent fatal SAE, the relapse episodes of the seven remaining patients could be controlled with oral 8 mg MP/day in combination with MTX (7.5 mg/week) and topical corticosteroids.

Seven of the 53 compliant patients experienced a serious adverse event (SAE) during follow up, leading to treatment discontinuation or substantial treatment modification; two of them died in the course of the SAE (respiratory infection in both cases). Clinical data of the SAE cases are summarized in Table 4. The overall SAE rate was 6.80 per 100 person-years [95% CI: 3.00–14.28] (Figure 4B). Core demographic and treatment outcome data did not differ between patients with and without an SAE (Table 5).

Of the seven confirmed SAE one occurred before the sixth month of follow-up and another concurrently with a relapse. Accordingly, 13 of the 44 patients experienced a feasibility-limiting event (either relapse or SAE) 6 months after the onset of the treatment, i.e., 17.63 [95% CI: 9.96–30.24] feasibility limiting events per 100 person-years. Nevertheless, approximately three-fourths (73.4 ± 7.9%) of these BP patients had suffered neither of the above adverse events at the three-year follow up (Table 3). Notably, according to a multivariate approach of the key patients’ characteristics, only the MP dose at week 12 of treatment was an independent, statistically significant predictor of a feasibility-limiting event: Patients who were off MP at week 12 of the treatment were at a higher risk of developing a feasibility limiting event 6 months or later after starting treatment (*p* = 0.013, Cox proportional hazards model; Table 6), which means that continuing on low-dose MP intake (≤8 mg/day) beyond week 12 significantly lowered the risk of a feasibility-limiting event thereafter. The association of either of the feasibility-limiting event (relapse or SAE) with the MP intake at week 12 reflects an increased risk of the patients who had already stopped MP intake to suffer a BP relapse (*p* = 0.002, Mantel–Cox test) without, however, respective modification of their SAE risk (*p* = 0.423, Mantel–Cox test; Figure 4, inserts, and Table 7). In further assessment of the optimal 12-week MP dose, no difference in relapse risk, in SAE risk, or in either of them between patients receiving, at that time, 4 mg or 8 mg MP (*p* = 0.897, *p* = 0.076, and *p* = 0.197 for relapse and SAE, respectively; Mantel–Cox test) could be identified.

**Figure 5:**
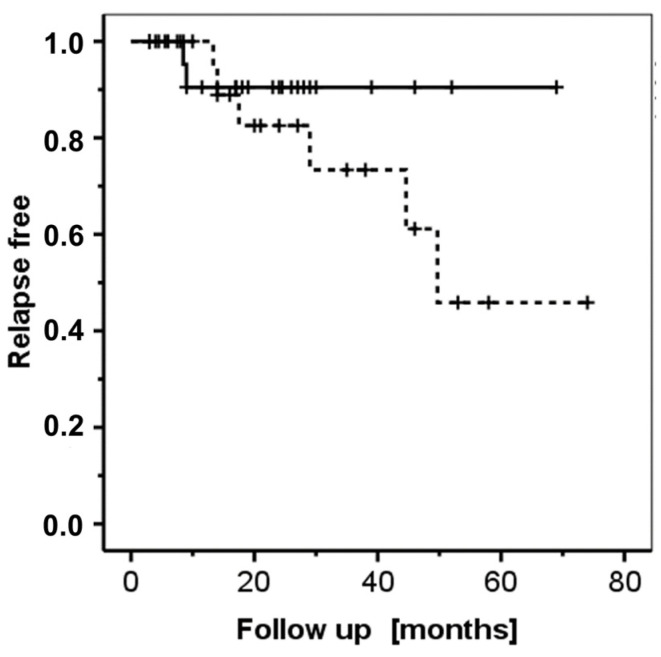
Relapse-free survival as a function of follow-up time (in months). Solid line: Gliptin (DPP4i)-related BP. Dashed line: non-gliptin-related BP. The short perpendicular bars represent censoring events. There is no significant difference in the times to relapse between the two patients’ groups (*p* = 0.257; Log Rank Mantel-Cox test).

Finally, it is worth noting that in the present cohort, DMT2 patients with DPP4i-associated BP seemed to be at a lower risk for relapse, given that the inflicting medication was discontinued after the diagnosis of BP; however, the difference was statistically not significant (*p* = 0.257, Log Rank Mantel–Cox test; Figure 5).

## 4. Discussion

With a *per intention to treat* remission rate of 96.4% at week 12 of treatment (*per protocol*: 100%), 29.5 days average time to remission, 80.7 ± 7.7% relapse-free patients at three-years follow up, and an acceptable SAE rate (6.80/100 py), the present combination of MP and MTX proved an efficacious regimen to initiate treatment in all patients with newly diagnosed BP. The low rate of disease relapses in this cohort (80.7 ± 7.7% relapse-free patients at three-years follow-up) is in line with the findings of the study by Κjellman and colleagues [8], who reported that the co-administration of MTX and glucocorticoids in a cohort of patients with BP, with comparable demographic characteristics to the herein presented, optimizes the treatment response and, especially, the long-term maintenance of remission. Notably, in the study by Kjellman and colleagues [8], the addition of MTX compared to monotherapy with glucocorticoids improved the rate of disease-free patients at the two-year follow-up; however, the achieved response rate was 65%, which is lower than that presently reported (86.4 ± 5.7%).

It is worth noting that the fraction of patients who remained disease-free five years after attaining remission (61.0 ± 14.0%, however, with only 2 patients still remaining in follow up; Figure 4A) was adequately high and generally equivalent to the 50% after the 5.5-year follow-up, which was reported with the co-administration of prednisone and MTX in patients with BP in the small study (*n* = 16 patients) of Kwatra and Jorizzo [20]. Despite the differences in the definition of endpoints, the latter study is comparable to ours in terms of the initial dosages of both glucocorticoids (20–60 mg prednisone/day vs. 16–64 mg methylprednisolone presently) and MTX (2.5–15.0 mg versus 2.5–10.0 mg presently). However, with an average daily methylprednisolone dose of 3.96 mg at the three-month follow-up (equivalent to about 5 mg prednisone), the rate of tapering the glucocorticoids in the present cohort seems to be faster compared to that anticipated from the halving of the initial dose of prednisone within six months of the initiation of treatment, reported in the study of Kwatra and Jorizzo [20].

Furthermore, in a recent study, BP treatment with low-dose MTX monotherapy (generally no more than 12.5 mg/week) proved an adequately efficacious therapeutic modality in patients with BP and impaired renal function [26]. However, the calculated 63.4% remission rate in the latter study seems to be inferior compared to the >90% in the present cohort. Notably, the presently 58-month median time to MTX discontinuation was much longer compared to the 459 days (~15 months) of the latter study.

Compliance to the treatment (96.36% or 53/55 of the patients) was also satisfactory, considering the age of the patients and the fact that adherence to a treatment generally decreases with increasing age and degree of treatment complexity [27,28,29].

The feasibility of the present combination for BP patients is also supported by the overall encouraging safety profile of the combination, as only 7/53 patients suffered a SAE over a follow-up period of many years (in average, 6.80 events per 100 person-years), and only two elderly patients died in the course of their SAE (3.77% of the cohort patients). Sparing of the cumulative glucocorticoid burden with the presently proposed combination modality, which corresponds to weekly and not to bi-weekly tapering steps, as suggested in BP treatment guidelines, might have contributed to this latter outcome. At this point, it is important to emphasize that the spectrum of the observed SAE in the present cohort could have been attributed to the use solely of systemic glucocorticoids.

Furthermore, our results point to a MP tapering strategy that enhances the feasibility of the proposed treatment approach for BP patients. An interesting observation of this study is that patients in whom MP had already been discontinued at week 12 of the treatment were at a significantly higher risk for a future relapse compared to those still receiving a low MP dose (≤8 mg). Notably, these latter patients were not at a higher risk of manifesting an SAE (*p* = 0.423). This suggests that the continuation of low-dose MP might improve the feasibility of the present combination. In practice, a relatively ‘safe’ MP tapering down to a daily dose of 8 mg may last <10 weeks for patients with a starting MP daily dose of 64 mg, <7 weeks for 48 mg, and just 3–4 weeks for 32 mg MP/day. The daily MP dose can be reduced thereafter to 4 mg and maintained at this level for a period still to be elucidated in future studies.

Finally, it is worth noting that BP seems to be controlled faster in the group of ‘older’ (≥75 years old) compared to the ‘younger’ (<75 years old) patients with the present therapeutic proposal (shorter ‘disease control time’ by about 11.5 days, *p* = 0.022). At the same time, no differences were observed in the feasibility measures of the treatment, especially in adverse reactions, findings that encourage the prescription of methotrexate in older patients with BP in whom it has been also suggested as a potential monotherapy. [14]

The main limitation of this study is the absence of a control arm treated exclusively with systemic glucocorticoids (MP), a consequence of its retrospective character. In addition, data of disease severity measurements were not available for many patients and were not included in this study.

## 5. Conclusions

In conclusion, as most of the cumulative glucocorticoid dose necessary to treat BP is administered within the first 6 months after treatment initiation [2] it is important to taper them promptly. Despite several limitations, our results are supported by evidence from comparable studies [8,14,16,21,26] and suggest that a therapeutic combination scheme of fast, weekly, methylprednisolone tapering plus a moderate methotrexate dose is a safe; efficient; and, most probably, glucocorticoid-sparing treatment modality for the initiation of the treatment of patients with a newly diagnosed bullous pemphigoid.

## Figures and Tables

**Table 1:** Core demographic and treatment parameters of the cohort (*n* = 53, ***Total***). In addition, a comparison of patients taking dipeptidyl peptidase 4 inhibitor (*n* = 24) or not (*n* = 29) at the time of bullous pemphigoid diagnosis.

Parameter	DPP4i ^a^	*n*	Mean	S.E. ^b^	95% CI ^c^ of Mean		*p* *
					Lower	Upper	
Age [years]	No	29	80.31	1.47	77.30	83.32	0.234
	Yes	24	77.46	1.88	73.57	81.35	
	***Total***	***53***	***79.02***	***1.18***	***76.66***	***81.38***	
Initial daily MP ^d^ dose [mg/day]	No	29	35.31	2.30	30.60	40.02	0.770
	Yes	24	35.33	1.92	31.36	39.31	
	***Total***	***53***	***35.32***	***1.52***	***32.28***	***38.36***	
12-week daily MP dose [mg/day]	No	29	4.00	0.77	2.43	5.57	0.828
	Yes	24	3.92	0.60	2.68	5.15	
	*****Total*****	*****53*****	***3.96***	***0.50***	***2.97***	***4.96***	
Cumulative MP dose to TDC ^e^ [mg]	No	29	679	78	517	840	0.791
	Yes	24	677	57	557	797	
	*****Total*****	*****53*****	***678***	***49***	***578***	***777***	
12-week cumulative MP dose [mg]	No	29	1037	79	875	1199	0.805
	Yes	24	971	66	833	1110	
	*****Total*****	*****53*****	***1009***	*****53*****	***902***	***1115***	
TDC [days]	No	29	30.29	3.50	23.11	37.46	0.894
	Yes	24	28.57	2.83	22.69	34.44	
	*****Total*****	*****53*****	***29.51***	***2.29***	***24.92***	***34.10***	
MP dose reduction rate [mg/week] ^f^	No	29	8.33	1.24	5.80	10.87	0.394
	Yes	24	8.18	0.80	6.51	9.84	
	*****Total*****	*****53*****	***8.26***	***0.76***	***6.73***	***9.80***	
Initial MTX ^g^ dose [mg/week]	No	29	6.55	0.26	6.02	7.09	0.070
	Yes	24	7.29	0.30	6.68	7.91	
	*****Total*****	*****53*****	***6.89***	***0.20***	***6.48***	***7.29***	
12-week MTX dose [mg/week]	No	29	6.72	0.45	5.80	7.64	0.258
	Yes	24	6.98	0.40	6.16	7.80	
	*****Total*****	*****53*****	***6.84***	***0.30***	***6.23***	***7.45***	

^a^ DPP4i: Dipeptidyl peptidase 4 inhibitor; ^b^ S.E.: standard error; ^c^ CI: 95% confidence interval; ^d^ MP: methylprednisolone; ^e^ TDC: time to disease control; ^f^ from treatment onset to week 12; ^g^ MTX: methotrexate. * Comparison of patients with and without DPP4i intake at the time of BP diagnosis: ANOVA. Average data of the cohort as a whole are indicated as ***Total*** in bold italics.

**Table 2:** Central parameters of the therapeutic response: Comparison between ‘younger’ (<75-years-old, *n* = 14) and ‘older’ (≥75 years old, *n* = 39) patients.

Parameter	Age	Mean	S.E. ^a^	95% CI of Mean ^b^		*p* *
				Lower	Upper	
12-week cumulative MP ^c^ dose [mg]	<75	1112	124	841	1383	0.259
	≥75	974	57	858	1090	
TDC ^d^ [days]	<75	37.93	5.87	25.24	50.62	0.022
	≥75	26.32	2.07	22.13	30.52	
Follow up [months]	<75	28.18	5.38	16.55	39.81	0.275
	≥75	21.97	2.78	16.33	27.62	
MP dose reduction rate [mg/week] ^e^	<75	7.22	1.28	4.45	9.99	0.407
	≥75	8.66	0.93	6.76	10.55	

^a^ S.E.: standard error; ^b^ CI: 95% confidence interval; ^c^ MP: methylprednisolone; ^d^ TDC: time to disease control; ^e^ from treatment onset to week 12. * Comparison of ‘younger’ vs. ‘older’ patients: ANOVA.

**Table 3:** Percentage (%) of event-free patients as a function of the follow-up time for relapses, severe adverse events, or either of latter events (Kaplan–Meier method).

	% Event-Free Patients [SE] ^b^		
Relapse OR SAE ^a^	12 Months (*n* = 34) ^c^	24 Months (*n* = 18)	36 Months (*n* = 10)
Either event [*n* = 44] ^d^	92.5 [4.2]	77.9 [7.0]	73.4 [7.9]
Relapse [*n* = 44]	95.1 [3.4]	86.4 [5.7]	80.7 [7.7]
SAE [*n* = 44]	97.3 [2.7]	94.5 [3.8]	84.0 [7.8]
SAE [*n* = 53]	95.2 [3.3]	92.5 [4.2]	82.2 [7.8]

^a^ SAE: severe adverse event; ^b^ SE: standard error; ^c^ *n*: number of patients still on follow up; ^d^ *n*: number of patients entered in the calculation (either all compliant throughout week 12 of treatment, *n* = 53, or only those with follow up ≥ 6 months, *n* = 44).

**Table 4:** Clinical data of patients with severe adverse events (SAE). Of note, all diabetes mellitus type 2 (DMT2) patients required insulin treatment because of glucocorticoid-induced deterioration of glycemic control.

Gender, Age	DMT2/History of DPP4i ^a^ Use	Comorbidities	Severe Adverse Event	Time from Diagnosis to SAE ^b^ [Months]	Clinical Course-Outcome
Female, 79	No/No	Hypertension, COPD ^c^, hyperlipidemia, epilepsy	Respiratory tract infection	53	Deceased
Female, 81	No/No	Hypertension, hypothyroidism	Hyponatremia	5	Stopped MP ^d^, treatment continued with topical steroids. Patient stopped MTX ^e^ by herself. Without new lesions at last follow-up visit (58 months since diagnosis).
Female, 87	Yes/Yes	Hypertension, hypothyroidism	Stroke	38	Stopped MP intake and reduction of MTX dose.
Male, 89	Yes/Yes	Hyperlipidemia, depression	Hip fracture	14	Stopped MP intake. Patient continued MTX (overall treatment duration: 12 months).
Female, 69	No/No	Hypertension, atrial fibrillation, rheumatoid arthritis, hypothyroidism, heterozygous beta thalassemia, GERD ^f^	Respiratory tract infection	25	Stopped MP and MTX.
Female, 90	Yes/Yes	Psoriasis, colitis ulcerosa, cataract, glaucoma	Stroke	26	Stopped MTX after the stroke. Without new lesions at last follow-up (28 months since diagnosis; 2 months after the SAE)
Female, 84	Yes/Yes	Hypertension, hyperlipidemia, GERD, osteoporosis	Respiratory tract infection	15	Deceased

^a^ DPP4i: dipeptidyl peptidase 4 inhibitor; ^b^ SAE: severe adverse event; ^c^ COPD: chronic obstructive pulmonary disease; ^d^ MP: Methylprednisolone; ^e^ MTX: methotrexate; ^f^ GERD: gastro-esophageal reflux disease.

**Table 5:** Comparison of selected core clinical parameters of the *n* = 7 patients with a severe adverse event (SAE) with those of the rest of the patients without development of an SAE.

Parameter	SAE ^a^	Mean	S.E. ^b^	95% CI ^c^ for Mean		*p* ^d^
				Lower	Upper	
Age [years]	No	78.1	1.25	75.6	80.6	0.177
	Yes	82.7	2.75	76.0	89.4	
12-week cumulative MP ^e^ [mg]	No	979	53	871	1087	0.157
	Yes	1201	204	703	1699	
12-week daily MP dose [mg]	No	3.92	0.34	3.26	4.61	0.051
	Yes	5.82	1.06	3.21	8.41	

^a^ SAE: severe adverse event; ^b^ S.E.: standard error of the mean; ^c^ 95% CI: 95% confidence interval; ^d^ *p*: ANOVA; ^e^ MP: methylprednisolone.

**Table 6:** Cox proportional hazards model of suffering at least one feasibility-limiting event (relapse or severe adverse event, the one that occurred first). Overall, 13/44 patients with a follow-up time ≥ 6 months developed a feasibility-limiting event.

Predictor	*p* ^a^	OR ^b^	95.0% CI ^c^ for OR	
			Lower	Upper
Gender (male vs. Female)	0.193	3.167	0.559	17.941
Age (<75 vs. ≥75 years)	0.057	0.121	0.014	1.061
Gliptin (intake at BP diagnosis: yes vs. no) ^d^	0.188	2.608	0.626	10.858
MP ^e^ (initial daily dose [mg])	0.139	1.036	0.989	1.086
MP (12-week daily dose: 0 mg vs. >0 mg) ^f^	0.013	8.726	1.572	48.439
Methotrexate (initial weekly dose [mg])	0.132	1.513	0.883	2.593

^a^ *p*: significance level; ^b^ OR: odds ratio; ^c^ CI: confidence intervals; ^d^ Half of the patients (49%) were co-morbid for diabetes mellitus type 2 (DMT2), with 92.3% of them on DPP4i treatment at the time of BP diagnosis. The core demographic and disease parameters did not differ significantly between patients with and without a history of DPP4i treatment. ^e^ MP: Methylprednisolone; ^f^ of the 44 patients with follow up ≥ 6 months, *n* = 13 were off MP (0 mg), *n* = 19 on 4 mg, and *n* = 12 on 8 mg at 12 weeks after treatment initiation.

**Table 7:** Event-free follow-up time for the *n* = 44 patients with follow-up time ≥ 6 months. Comparison between patients still taking methylprednisolone (MP) or not at week 12 of treatment.

Adverse Event	12-Week MP ^a^ Dose [mg/Day]	Event-Free Follow-Up Time [Months]				*p* ^d^
		Mean	S.E. ^b^	95% CI ^c^		
				Lower	Upper	
Relapse OR SAE ^e^	0	29.12	4.78	19.76	38.48	0.002
	>0 ^f^	56.60	5.44	45.93	67.27	
Relapse	0	36.14	5.06	26.23	46.06	0.003
	>0	69.34	3.17	63.12	75.56	
SAE^5^	0	39.18	4.22	30.91	47.45	0.423
	>0	60.00	5.83	48.58	71.42	

^a^ MP: methylprednisolone; ^b^ S.E.: standard error; ^c^ CI: confidence intervals; ^d^ *p*: significance by Log-rank test; ^e^ SAE: severe adverse event; ^f^ either 4.0 or 8.0 mg/day.

## Data Availability

Not applicable.
